# Genetic and Epigenetic Control of *CDKN1C* Expression: Importance in Cell Commitment and Differentiation, Tissue Homeostasis and Human Diseases

**DOI:** 10.3390/ijms19041055

**Published:** 2018-04-02

**Authors:** Emanuela Stampone, Ilaria Caldarelli, Alberto Zullo, Debora Bencivenga, Francesco Paolo Mancini, Fulvio Della Ragione, Adriana Borriello

**Affiliations:** 1 Department of Precision Medicine, University of Campania “Luigi Vanvitelli”, 80138 Naples, Italy; ema.stampone@gmail.com (E.S.); ilariacaldarelli@libero.it (I.C.); deborabencivenga@yahoo.it (D.B.); 2 Department of Sciences and Technologies, University of Sannio, 82100 Benevento, Italy; albzullo@unisannio.it (A.Z.); mancini@unisannio.it (F.P.M.); 3 CEINGE Biotecnologie Avanzate S. C. a R. L., 80145 Naples, Italy

**Keywords:** p57^Kip2^, CDKN1C, epigenetics, disease, cell differentiation

## Abstract

The *CDKN1C* gene encodes the p57^Kip2^ protein which has been identified as the third member of the CIP/Kip family, also including p27^Kip1^ and p21^Cip1^. In analogy with these proteins, p57^Kip2^ is able to bind tightly and inhibit cyclin/cyclin-dependent kinase complexes and, in turn, modulate cell division cycle progression. For a long time, the main function of p57^Kip2^ has been associated only to correct embryogenesis, since *CDKN1C*-ablated mice are not vital. Accordingly, it has been demonstrated that *CDKN1C* alterations cause three human hereditary syndromes, characterized by altered growth rate. Subsequently, the p57^Kip2^ role in several cell phenotypes has been clearly assessed as well as its down-regulation in human cancers. *CDKN1C* lies in a genetic locus, 11p15.5, characterized by a remarkable regional imprinting that results in the transcription of only the maternal allele. The control of *CDKN1C* transcription is also linked to additional mechanisms, including DNA methylation and specific histone methylation/acetylation. Finally, long non-coding RNAs and miRNAs appear to play important roles in controlling p57^Kip2^ levels. This review mostly represents an appraisal of the available data regarding the control of *CDKN1C* gene expression. In addition, the structure and function of p57^Kip2^ protein are briefly described and correlated to human physiology and diseases.

## 1. Introduction

A well-orchestrated sequence of events allows the transition between the various phases of cell division cycle and the precise control of a perfect execution and accomplishment of each phase. Central actors in this process are heterodimers formed by cyclin/cyclin-dependent kinase complexes (CDK) whose activity is strictly regulated by a number of factors, including their amount, localization, and post-synthetic modifications (mainly phosphorylations). A further important modulation is due to the interaction with additional inhibitory proteins resulting in the formation of heterotrimers, generally lacking the kinase activity. These proteins are defined CDK inhibitors (CKI) or, alternatively, CDK regulator. One family of CKI, established on the basis of sequence homology and specificity of action, is named CIP/Kip and includes three members, namely p21^Cip1/WAF1^, p27^Kip1^ and p57^Kip2^. Due to their broad inhibitory effect on cyclin-CDK complexes, CIP/Kip members have been mainly considered as antiproliferative proteins and their encoding genes as potential tumor suppressor genes. However, strong emerging pieces of evidence have demonstrated that the activities of CIP/Kip members are well beyond that of modulators of cell division [1]. Indeed, in function of their localization and interactors, CIP/Kip members might regulate a plethora of events including cell differentiation, cell movement, apoptosis, autophagy and all the major steps of carcinogenesis [1]. In addition, the tissue-specific functions of p57^Kip2^ cannot be substituted by other CIP/Kip family members, suggesting that each of them has peculiar roles in cell physiology.

In this brief review, we provide an appraisal of the published data on the p57^Kip2^ protein, that represents the least studied member within the CIP/Kip family. Our attention will be mainly focused on the regulation of *CDKN1C* (the p57^Kip2^ encoding gene) expression and its relevance in human diseases, including overgrowth and undergrowth syndromes.

### 1.1. p57^Kip2^ Protein

Human *CDKN1C* encodes a 316-amino-acid protein that migrates at 57 kDa by SDS-PAGE electrophoresis, hence the name p57^Kip2^. p57^Kip2^ is the last identified member of the CIP/Kip family of the cyclin-dependent kinase inhibitors, also including p21^Cip1/WAF1^ and p27^Kip1^ [2,3]. The CIP/Kip proteins share structural similarity mainly related to the common activity of cell cycle regulators. The most characterized Cyclin/CDK inhibitory activity relies on two common features: a CDK binding/inhibitory domain (KID) located at the amino-term and the nuclear localization signal (NLS) at the carboxy terminal of the protein [4]. The KID includes three short peculiar motifs: a cyclin-binding domain, a CDK-binding site and a 3_10_ helix that, due to a specific pair of amino acids (phenylalanine-tyrosine), is able to mimic the adenine component of ATP, therefore blocking the catalytic site of CDKs [5]. Similarly to other CIP/Kip members, KID is necessary and sufficient to bind and inhibit CDK activity. Specifically, it has been reported that p57^Kip2^ inhibits the kinase activity of cyclin-CDK complexes in vitro, including cyclin E (A)/CDK2 and cyclin D1,2/CDK4 [2,3,6]. Besides CDKs, several other proteins have been reported to interact with the p57^Kip2^ amino-terminal domain. Particularly, known interactors of p57^Kip2^ at its N-terminal domain are the basic helix-loop-helix transcription factors, such as MyoD, Mash1, NeuroD, and Nex/Math2 [6,7,8]. Furthermore, p57^Kip2^ interacts, both in vivo and in vitro, with the transcription factor B-Myb, which plays an important role during early embryonic development. Particularly, B-Myb competes with cyclin A2 for binding to p57^Kip2^, thus determining the release of active cyclin/CDK2 [9].

The carboxy-terminal region of p57Kip2 contains a QT box domain, rich in glutamine and threonine residues, which is homologous to the corresponding QT domain of p27^Kip1^ and can be responsible for further interactions of the protein. It has been reported that the QT box directly binds to c-Jun NH2-terminal kinase/stress-activated protein kinase, determining its inhibition [10,11]. In the QT domain, a consensus sequence for a putative nuclear localization signal (NLS) has been identified [2,3]. Proceeding towards the C-terminal, p57^Kip2^ presents, in homology with p21^Cip1/WAF1^, a binding domain for the proliferating cell nuclear antigen (PCNA), a cofactor of DNA polymerase delta. Thus, it is able to bind and inhibit PCNA, even though with much lower affinity than p21^Cip1/WAF1^ [12].

Whereas the p57^Kip2^ amino- and carboxy-terminal domains are similar in sequence in mammals, the internal domain, consisting of proline/alanine-rich motifs, results as a peculiarity of human p57^Kip2^: the PAPA region, a sort of hinge between the N- and the C-end of the protein. It is absent in p21^Cip1/WAF1^ and p27^Kip1^ and is responsible for the difference between the sequence-derived molecular weight and the SDS-PAGE observed molecular weight. The PAPA region is scarcely conserved in mouse and rat, where it is substituted by a proline-rich region followed by an acidic repeat in which glutamic or aspartic acid occur every four amino acids [3]. However, the functional meaning of the PAPA region is still unknown, even though some authors retain that it is important for protein interactions.

A peculiar characteristic of p57^Kip2^ protein is a limited degree of stable secondary and tertiary structures under physiological conditions. Specifically, the protein belongs to the so-called intrinsically unstructured proteins (IUPs), which can adopt different conformations upon binding to distinct and specific interactors. This property is shared with its siblings p21^Cip1/WAF1^ and p27^Kip1^, and with numerous proteins involved in the control of cell proliferation. As a matter of fact, more than 70% of human cancer-associated proteins are IUPs. This conformational flexibility allows a considerable versatility in terms of biomolecular interactors, expanding the range of their functions and, in turn, their involvement in numerous cellular processes [1]. On the other hand, post-synthetic changes of an IUP, like (but not only) phosphorylations, might play a fundamental role in guiding the protein towards specific interactions and specific functions. So far, only few phosphorylation sites have been identified in human p57^Kip2^ protein, such as threonine 310 (T310). Particularly, T310 phosphorylation has been suggested as being important for human protein degradation [1] and level control. Specifically, in analogy with p27^Kip1^ threonine 187 phosphorylation [13], the phosphorylation on T310 determines a phosphodegron which functions as a recognition site for the substrate recognition subunit (S-phase kinase-associated protein 2, Skp2) of the E3 ubiquitin ligase SCF complex (Skp1/Cul1/F-box protein). The Skp2-SCF complex guides target proteins to proteasomal degradation in a cell cycle-dependent manner (from late G1 to early M phase) and its activity appears strongly deregulated in human cancers [12]. Furthermore, besides the Skp2-SCF complex, the activity of the SCF-FBL12 complex, whose substrate recognition subunit (FBL12) is different from Skp2, has been reported to be involved in TGFβ1-induced p57^Kip2^ ubiquitin-dependent proteasomal degradation in osteoblast cells [14].

### 1.2. p57^Kip2^ in Embryonic and Adult Tissues

p57^Kip2^, unlike the other two CKIs, shows a fine-tuned temporal and spatial expression from embryogenesis up to the adult life. p27^Kip1^ and p57^Kip2^ are widely expressed during embryogenesis. p27^Kip1^ is more abundant in ovary, testis, thymus, spleen and developing retina, instead, p57^Kip2^ is mostly localized in cartilage, skeletal muscle, palate, pancreas, and intestine. Interestingly, the CKIs show a complementary expression pattern in several embryonic areas. Indeed, in the adrenal gland, p27^Kip1^ is only expressed in the medulla, while p57^Kip2^ is exclusively found in the cortex [2,3,15,16]. In contrast, p21^Cip1/WAF1^ is highly expressed in terminally differentiated cells of adult tissues rather than in embryonic cells [17,18], except for the embryonic carcass where there is an extensive muscle differentiation [18,19,20].

In adult tissues, p21^Cip1/WAF1^ and p27^Kip1^ are widely expressed, whereas p57^Kip2^ is detectable only in a restricted subset of mouse and human tissues/organs, such as placenta, fat, kidney, ovary, adrenal gland, endometrium, lung, prostate, brain, kidney, pancreas, testis, heart and skeletal muscle [2,3,16]. In most tissues, p57^Kip2^ is expressed at a low level. This may reflect the heterogeneity of some of these tissues where only certain cell types express the protein. The highest expression level is found in human placenta, particularly in the villus section of placenta, together with other genes involved in growth and tissue remodeling, like IGF2 and GPC3 [21]. In mice, during placental development, p57^kip2^ is expressed in giant trophoblast cells. Therefore, p57^Kip2^ has been postulated to be involved in the allocation of maternal nutrients through the placenta [22,23]. Since human placenta lacks a cell type equivalent to the giant trophoblast cell, the function of p57^Kip2^ in human placenta might be different and further investigations appear necessary [24].

p57^Kip2^ level declines, in most organs, before birth, whereas p27^Kip1^ expression persists after birth and throughout adult life, suggesting that p57^Kip2^ is important during early organogenesis [15]. The crucial role of p57^Kip2^ in embryogenesis is corroborated by the finding that *CDKN1C* knockout mice (p57^KO^) die after birth with only less than 10% of the mutant mice surviving until weaning. p57^KO^ mice show severe defects such as macroglossia, cleft palate, omphalocele and gastrointestinal abnormalities, skeletal muscle and endochondral ossification defects, adrenocortical hyperplasia, lens cell hyperproliferation and apoptosis [25,26]. p57^KO^ mice also present several placental abnormalities, including trophoblastic dysplasia [27,28]. Conversely, p21^KO^ mice develop normally [29,30] and p27^KO^ mice do not present gross developmental defects, even though the protein is expressed during embryogenesis and it is required for development. However, p27^KO^ mice display organ hyperplasia and tumorigenesis, consistent with the expected function of inhibitor of cell proliferation [31,32,33]. The importance of a proper control of p57^Kip2^ dosage is also evident in mice that express a twofold level of p57^Kip2^. They show an increase of embryonic lethality and a decreased body size [25,28]. Furthermore, the replacement of *Cdkn1c* with *Cdkn1b* (p57^KO^p27^KI^) cannot completely compensate for the specific role of p57^Kip2^. In general, p27^Kip1^knock-in corrected many of the abnormalities observed in p57^KO^ mice, except for omphalocele, dysplasia of placenta and renal papilla [34]. This evidence supported the opinion that most of the functions performed by both p27^Kip1^ and p57^Kip2^ proteins during development are attributable to the CKI role through their conserved N-terminal KID domain. Thus, the phenotypic differences noticed in p27^KO^ and p57^KO^ mice most probably reflect both their different spatiotemporal expression patterns and the diverse cellular behavior towards an incomplete cell cycle inhibition [34]. However, it is also possible that the C-terminus domain of both CKIs plays similar functions or affects superimposable pathways. In addition, it should be taken into consideration that p57^KO^p27^KI^ mice express non-physiological levels of p27^Kip1^.

In adult tissues, all the three CIP/Kip proteins are specifically expressed in terminally differentiated cells, but, of great interest, also in certain undifferentiated quiescent stem cells, probably because of their CKI activity. The finding that most p57^KO^ mice die soon after birth represented an obstacle for the characterization of p57^Kip2^ function in adult tissues. This issue has been overcome by the generation of conditional KO mice. So far, the tissue-specific deletion of *Cdkn1c* has been performed only in adult hematopoietic stem cells (HSCs) and in neural stem cells (NSCs), evidencing the pivotal role of p57^Kip2^ in the quiescence and maintenance of adult stem cells [35,36].

Among hematopoietic cell populations, p57^Kip2^ is the only CKI to be prevalent in a pool of cells with long-term repopulating capability [37] and hematopoietic-specific ablation of p57^Kip2^ in adult mice determines a clear depletion of the HSC population [35]. On the contrary, p21^Cip1/WAF1^ seems to be mainly important in regulating HSC cell cycle during stress condition when DNA is damaged [38,39]; instead, p27^Kip1^ has limited activities, but becomes more effective in later committed progenitors [38]. In vitro experiments partially confirmed the in vivo observations. High p57^Kip2^ mRNA and protein expression have been reported in the HSC side population, especially in c-kit(+)/Sca-1(+)/Lineage-SP cells and p57^Kip2^ has been designated as responsible for the cell cycle blockage since its downregulation is required for S phase entry [37,40]. Moreover, RNA-sequencing analysis of HSC populations derived from a mouse model with a lacZ knock-in at *Mds1* and *Evi1* complex locus, which eliminates the ME domain, has revealed the silencing of p57^Kip2^ expression and it is correlated with the reduction in the number of HSCs and a complete loss of long-term repopulation capacity [41]. Similar pieces of evidence have been obtained later by analyzing CKIs activities in NSCs. p21^KO^ and p27^KO^ mice show an increased proliferation of intermediate progenitor cells rather than of NSCs in the dentate gyrus of the hippocampus, where the two CKIs are barely expressed [42,43]. In contrast, p57^Kip2^ is abundant in NSCs and its expression decreases when these cells become committed and proliferative. Conditional deletion of p57^Kip2^ resulted initially in a transient recruitment of NSCs into the cell cycle, thus activating neurogenesis in brain of both young and aged mice, and later in an excessive depletion of the quiescent NSC population and impairment of hippocampal neurogenesis [36]. The new “disposable stem cell model” proposed recently by Encinas, might explain this phenomenon. During youth, the generation of new neurons is abundant in brain and progressively decreases with age. NSCs, upon activation, asymmetrically divide for limited rounds and then terminally differentiate into astrocytes, thus, dramatically reducing the pool of NSCs [44].

In vitro experiments reveal a dual role of p57^Kip2^: one is related to the division capability of adult stem cells and the other one to differentiation. Indeed, p57^Kip2^ mRNA and protein have been reported to be increased during differentiation of cerebral cortical precursor [45], oligodendrocytes [46], keratinocytes [47,48], podocytes [49] and skeletal myoblasts [50,51,52]. Skeletal muscle has a certain regenerative potential, given the presence of the satellite cells, which are muscle progenitor cells that become activated following muscle injury, thus progressing through self-renewal, proliferation, differentiation, and fusion with pre-existing mature muscle fibers to replenish the lost muscle tissue [53]. In skeletal muscle cells, p57^Kip2^ participates in the balancing of progenitor cell maintenance with muscle differentiation [54]. Indeed, *Cdkn1c* is upregulated in murine G_0_ muscle satellite cells, and its inhibition is needed for satellite cell proliferation [55,56].

Data supporting a possible functional repair of the cardiac tissue have been accumulated over the last decades [57]. Indeed, this hypothesis relies also on the presence of cardiac progenitor cells, named cardiac stem cells (CSCs). The block of cell cycle progression in murine c-kit+ CSCs is due to a complex signaling which involves also the upregulation of *Cdkn1c* [58]. Moreover, experimental evidence in mice demonstrated that cell cycle withdrawal in neonatal cardiomyocytes is associated with an increased expression of p57^Kip2^, p21^Cip1/WAF1^, and p27^Kip1^ and that in adult cardiomyocytes, silencing CDK inhibitors, including p57^Kip2^, induces cell cycle re-entry [59,60,61]. In addition, studies in transgenic mouse reported a cardioprotective effect of ventricular-specific overexpression of p57^Kip2^ with no side-effects on heart development [62]. Interestingly, also studies on zebrafish demonstrated that the repression of p57^Kip2^ expression promotes heart regeneration [63].

Importantly, several pathways have been reported to modulate the expression of p57^Kip2^ [64]. TGFβ/Smad signaling upregulates p57^Kip2^ expression in HSCs, mediating the maintenance of hematopoietic stem cells [65], while it has been reported to induce p57^Kip2^ degradation in osteoblasts [14]. On the contrary, Wnt/β-catenin and Notch/Hes pathways are reported to reduce p57^Kip2^ expression in several cell types. For example, in midbrain dopaminergic neurons, Wnt1 downregulates p57^Kip2^ [66], in lens epithelium [67] and in pancreas [68] Notch effectors suppress p57^Kip2^ expression. However, the general picture is complex and difficult to understand due to the cross-talk and overlapping of different signal pathways.

### 1.3. CDKN1C Mapping and Structure

**Figure 1:**
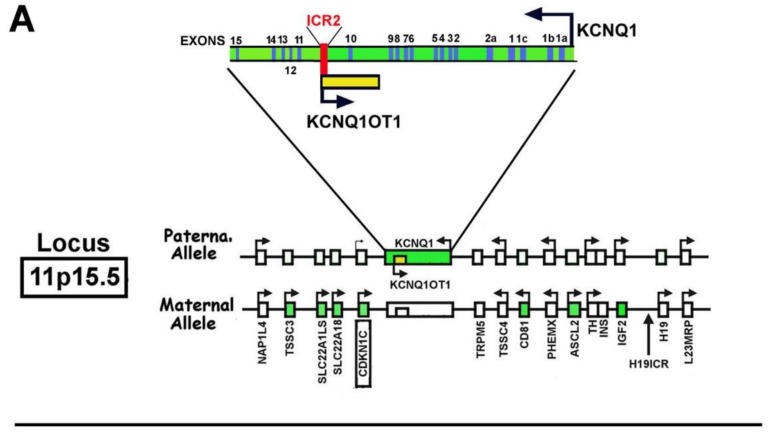

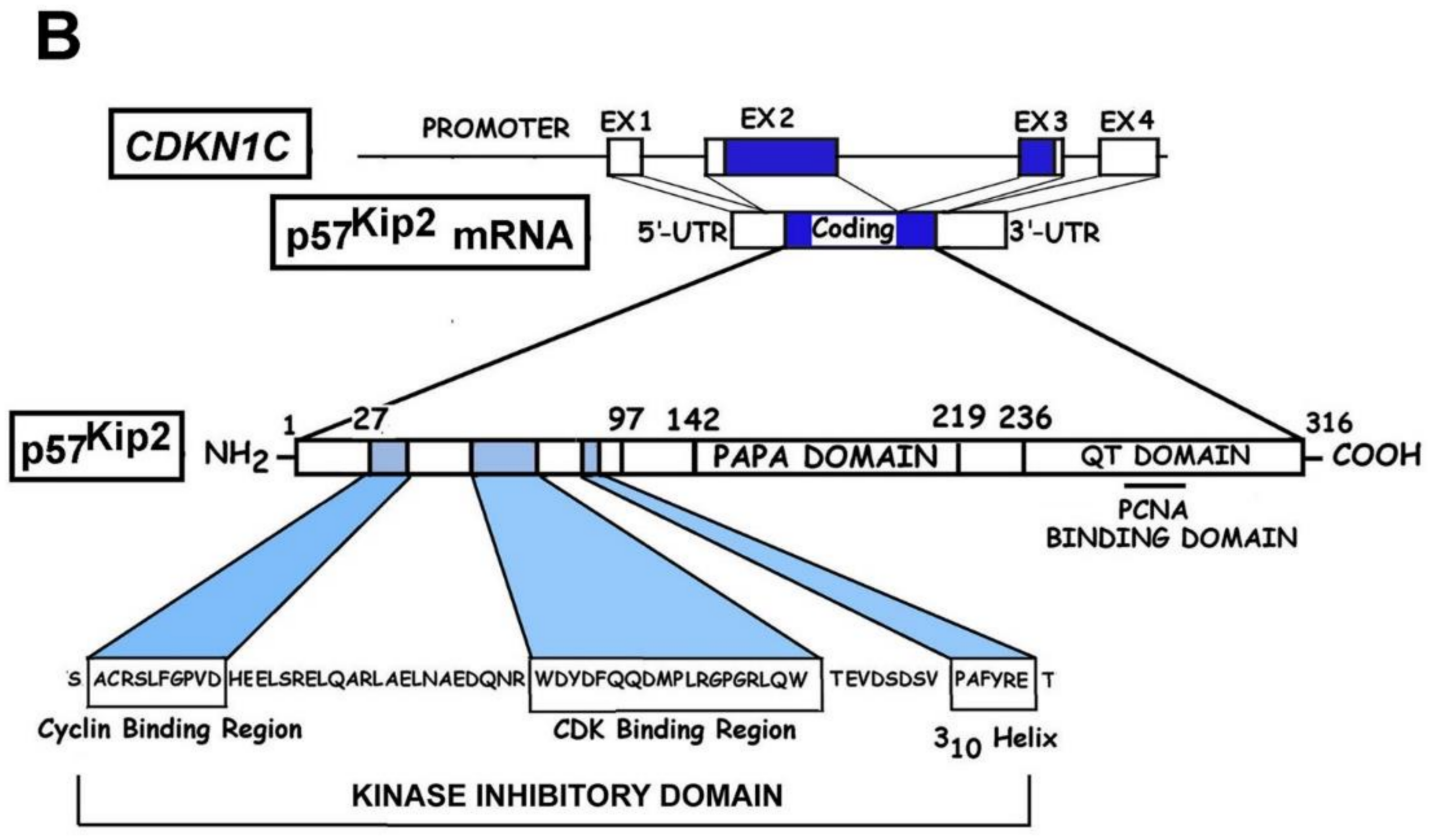
Structure of human 11p15.5 locus, *CDKN1C* gene, p57^Kip2^ mRNA and p57^Kip2^ protein. Panel (**A**) The panel shows the structure of the 11p15.5 locus with details of the *KCNQ1* exon organization (in blue boxes). *KCNQ1OT1* gene is included in the *KCNQ1* gene and transcribed in a different direction. The ICR2 region is shown in orange; Panel (**B**) The figure shows the structure of *CDKN1C* gene and p57^Kip2^ mRNA. In addition, at the bottom of the figure, it is represented the domain organization of p57^Kip2^ protein and the sequence of the KID (kinase inhibitory domain).

*CDKN1C* is localized, in humans, at the 11p15.5 locus and includes four exons and three introns (Figure 1). *CDKN1C* alternative splicing results in the formation of three mature mRNAs that have the same open reading frame, but different untranslated regions [69,70]. Human 11p15.5 locus contains numerous genes subjected to an imprinting modulation (Figure 1).

Importantly, the homolog region in mouse (i.e., the distal region of chromosome 7) shows an equal cluster of linked genes, arguing for the significance of their coordinate regulation and for the presence of maintained regulatory mechanisms [71,72].

The human 11p15.5 gene cluster might be divided into two distinct domains, both presenting a specific “*in cis*” acting ICR (Imprinted Control Region). The centromeric domain of the cluster is 800 kb long and is controlled by ICR2. The domain includes, in addition to *CDKN1C*, *KCNQ1* (*KvLQT1* or potassium voltage-gated channel, KQT-like subfamily member 1), *KCNQ1OT1* (also known as *LIT1*, *KCNQ1*-overlapping transcript 1 or long QT intronic transcript 1), *PHLDA2* (Pleckstrin homology-like domain family A member 2) and *SLC22A18* (Solute carrier family 22 member 18).

Structurally, ICR2 maps inside *KCNQ1* intron 10 and is methylated on the maternal chromosome; it encompasses the promoter for the non-coding RNA Kcnq1ot1 (antisense to *KCNQ1*) (Figure 1) [73].

ICR1 is telomeric and regulates the imprinting of *H19* (a gene for a long noncoding RNA) and *IGF2* (encoding for insulin-like growth factor 2) by restricting the access to the enhancers (i.e., ICR1 is a chromatin insulator) [74]. Interestingly several of these genes have distinct imprinting. Indeed, *IGF2* is paternally expressed, *H19* is maternally transcribed, and *CDKN1C* is maternally expressed, even though a weak expression of the paternal allele has been demonstrated in some human tissues [75].

Two main promoter elements have been identified in mouse *Cdkn1c* that are similar in humans. First, a proximal promoter element (−165 to +15 from the transcriptional start site) contains several *consensus* sequences for Egr1 and Sp1 [52,76]. Intriguingly, both transcription factors are ubiquitously expressed and have been reported to regulate other members of the CIP/Kip family of CDK inhibitors [77,78]. Furthermore, a binding site for GATA2, a transcription factor playing a pivotal role in hematopoiesis, particularly in early and late stages of erythropoiesis, and in the TGF-β-response has also been described [79]. Finally, this promoter region also contains recognition sequences for the transcriptional repressors CTIP2/Bcl11b, implicated in the developmental process and carcinogenesis, and the T-box transcription factor TBX3, which is involved in the tissue patterning and differentiation during embryonic development and is up-regulated in a plethora of cancers [80]. Importantly, the accessibility of the reported transcriptional modulators to the *CDKN1C* promoter is strongly influenced by the high presence of CpG islands, located upstream and downstream of the transcription start site, responsible for genomic imprinting and epigenetic gene silencing. This is achieved by CpG dinucleotide methylation and/or through chromatin remodeling by histone covalent modifications (histones H3 and H4 methylation and acetylation) [81]. More distal promoters have also been identified. They embrace E-boxes or E-box-like motifs for the interaction with basic-HLH proteins, including activators, like TCF4/E2-2 [82], E47 [83], Smad1/Atf2 complex [84], repressors, as Hes1 (a Notch effector) that, in intestinal crypt progenitor cells, inhibits *Cdkn1c* transcription by binding to a site located at −3300 [85], or Hes-related repressor protein Herp2 that acts as transcriptional repressor of *CDKN1C* in proliferating lens epithelial cells [67]. Furthermore, a glucocorticoid response element is located 5076 to 5062 bases upstream of the transcription start site of the human *CDKN1C* gene and is responsible for the glucocorticoid inducibility of the *CDKN1C* gene [86], thus explaining, at least in part, the antiproliferative effect of dexamethasone in human tumor cells such as Hela cell line [87].

In mouse, additional key elements of *Cdkn1c* transcription are located distantly from the gene. As a matter of facts, enhancers for its expression lie more than 25 kb downstream of the gene. Experiments with artificial chromosome also suggest the existence of enhancer(s) located very distantly from *CDKN1C* [88]. Accordingly, in humans, it has been suggested the presence of numerous *CDKN1C* enhancer elements localized in a region between 255–387 kb [89].

## 2. Control of *CDKN1C* Transcription

*CDKN1C* lies in humans and mice in a very complicated cluster of imprinted genes, controlled through superimposed *cis*-acting mechanisms. Genomic imprinting is an epigenetic process that results in parent-of-origin specific allelic expression [90]. A relatively small subset of genes within the mammalian genome (0.4%) is imprinted [91,92] showing a mono-allelic expression either in specific phenotypes of the whole organism or in peculiar tissues that favors the maternal (e.g., *CDKN1C* and *UBE3A*) or the paternal allele (e.g., *DLK1* and *NNAT*) [93]. Imprinted expression is initially determined by differential DNA methylation that is established in the germline [94].

Regarding *CDKN1C*, its transcription is regulated by the imprinting center KvDMR1 that acquires DNA methylation in the maternal germline [69,95,96]. This differentially methylated region spans the promoter of the paternally expressed long non-coding RNA Kcnq1ot1 required for continuous domain-wide imprinting. The *CDKN1C* promoter and gene body are also directly methylated on the paternal allele post-fertilization, after allelic silencing has been established [97].

Besides *cis*-acting mechanisms responsible for the imprinted silencing of the paternal allele (briefly summarized in the previous paragraph), *trans*-acting mechanisms participate in the epigenetic modulation of *CDKN1C* gene expression [98]. Indeed, a complex interplay among DNA methylation and post-translational modifications of histones contributes to the chromatin dynamics at the promoter and in *CDKN1C* gene body.

### 2.1. DNA CpG Island Methylation

*CDKN1C* gene is included in a CpG island extended about −600 bp from the transcriptional start site up into the gene body. This CpG island presents, in mice but not in humans, a differential methylation between the two inherited alleles, being the paternal one hypermethylated and the maternal one hypomethylated. This methylation pattern seems to be acquired successively to the ICR2-dependent DNA-modifications and is involved in the maintenance and reinforcement of the imprinted repression of the paternal allele. Several regulators have been involved in this process. One of them is Lsh (lymphoid-specific helicase) a protein belonging to the family of SNF2/helicases that act as chromatin remodeler and regulate DNA methylation. Lsh directly binds to *CDKN1C* promoter and allows the maintenance of hypermethylation of the paternal allele [99].

Complete biallelic hypermethylation occurs in human tumors and tumor cell lines [100,101], as well as in some undifferentiated tissues and cell types such as skeletal myoblasts. In this cell model, the activation of the transcription factor MyoD drives DNA demethylation on the maternal allele, therefore allowing the Myo-d-dependent expression of p57^Kip2^ [102].

Most interesting is also the role played by different members of the DNA methyltransferase (DNMT) family, the enzymes catalyzing the transfer of methyl groups to cytosines. Results from genetic ablation studies support the notion that not only DNMT1, mostly in charge of maintaining the methylation pattern of CpG islands during DNA replication, but also DNMT3a which is generally involved (together with DNMT3b) in de novo methylation of most imprinting control regions in the germline, are involved in *CDKN1C* promoter methylation. As matter of fact, both DNMT1 [56,103], and DNMT3a [56] have been found associated with *CDKN1C* promoter. Consistently with the importance of DNA methylation not only in paternal allele imprinting but also in p57^Kip2^ expression modulation in specific cellular and cell cycle phase contexts, treatment of many human tumor cell lines with demethylating agents such as 5-azacytidine and 5-aza-2′-deoxycytidine results generally in p57^Kip2^ expression activation [102,104].

### 2.2. Histone Marks

Histone modifications represent fundamental factors involved in chromatin plasticity, controlling gene promoter accessibility and gene expression activation [105,106].

Acetylation and methylation of core histone tails, in addition to DNA methylation, are key mechanisms for regulating *CDKN1C* transcription. Accordingly, the level of H3 and H4 acetylation directly correlates with the gene expression and, in turn, with several phenotypes including differentiation and carcinogenesis.

Specifically, a decrease of H3 lysine 4 dimethylation and histone H3 lysine 9 and 14 acetylation is observed on the paternal allele respect to the maternal one, facilitating its inactivation [107,108]. On the other hand, histone acetylation results to be increased on the paternal locus at the level of KvDMR1, following the expression of the long non-coding RNA and the corresponding *CDKN1C* inactivation [81].

Under various conditions, a direct correlation between *CDKN1C* expression and H3K9/K14 acetylation has been demonstrated. For example, cancer cells with low (or absent) p57^Kip2^ present histone hypoacetylation and, vice versa, tumors with a high level of the CKI show hyperacetylation [109,110]. These findings are confirmed by the re-expression of p57^Kip2^ after histone deacetylase (HDAC) inhibitor treatment [76]. Mechanistically, these changes involve the binding of HDACs, mostly HDAC1 and HDAC2, to the *CDKN1C* promoter region. We must underline that HDAC1 is highly expressed in many cancers including gastric [111], colorectal [112], hepatic [113], breast [114], and pancreatic cancer [115]. HDAC2 has been found mutated in colon cancer [116] and is overexpressed in esophageal [117], prostate [118], and gastrointestinal carcinomas [119].

An additional recognized histone epigenetic mark includes a lysine trimethylation, specifically H3K27me3 (trimethylation of lysine 27 of histone H3). Such a modification, also responsible for the paternal allele exclusion, is involved in the maturation of glial cells [120].

This histone trimethylation mark is due to the Polycomb repressive complex 2 (PRC2). The increase of H3K27me3 reduces *CDKN1C* expression, while its decrease, due to a reduction in the levels or activity of EZH, a specific promoter-binding PRC2 subunit, up-regulates gene transcription [121].

Di- and trimethylation of lysine 9 of the histone H3, an additional histone modification, also appears to control the expression of *KvDMR1* on the maternal locus, while it is not present on the paternal allele, in accord with the imprinted silencing of the paternally-derived allele [107,108].

In the same context, it is important to stress the role of histone modifications in MyoD control of *CDKN1C* expression. As matter of fact, an altered accumulation of H3K9me2 on the maternal KvDMR1 allele results in the lack of response to MyoD in that it reduces the accessibility of the transcription factor to the DNA [102].

In brief, the regulation of *KvDMR1* due to epigenetic factors (methylation of DNA, acetylation/methylation of histones) appears a key mechanism in the control of *CDKN1C* expression. The last several years have identified the existence of a strict crosstalk between all the epigenetic modifications, including the binding of modifying enzymes to the specific sites of action. In this complex interplay, an important role for non-coding RNAs has emerged.

### 2.3. LncRNA Involvement in Epigenetic Regulation

As for many genes playing fundamental roles in development, *CDKN1C* gene expression is controlled by lncRNAs, which act in strict crosstalk with signal pathway-induced transcription factors and chromatin modifiers, accounting for spatial- and temporal-specific gene activation during development or cell commitment and differentiation in adult life. Specifically, the macro lncRNA Kcnq1ot1, first discovered both in humans and mice as a KvDMR1-associated RNA, has emerged as a critical regulator of the chromatin status of the gene, at least in relation to the imprinting control [122]. *KCNQ1* and *KCNQ1OT1* share a region of overlapping DNA and are transcribed in opposite directions. *KCNQ1* encodes the potassium voltage-gated channel subfamily Q member 1, a protein required for the repolarization phase of the cardiac action potential. Differently from *KCNQ1* that allows the synthesis of a protein, *KCNQ1OT1* codifies a long-noncoding RNA that regulates the expression of several genes. Its promoter is normally hypermethylated in the maternal allele, thus hampering its expression. On the contrary, the paternal allele is normally transcribed, being hypomethylated [72,123]. When expressed, the long non-coding RNA remains in the nucleus where it is able to act on its own chromosome (i.e., it acts on chromatin *in cis*). Mechanistically, it is able to interact with histone methyltransferase complexes (like G9a, Suz12, and Ezh2) causing the enrichment of repressive histone modifications. This activity results in the epigenetic inactivation of paternally inherited *CDKN1C* [124]. Kcnq1ot1 is also able to bind DNMT1, allowing the hypermethylation of *CDKN1C* promoter and therefore implementing the repression of gene expression [103].

Additional long non-coding RNAs, like Tug1 (taurine upregulated gene 1) [125], Linc00668 [126] and HEIH-coding RNA [127] have been reported to modulate *CDKN1C* expression. Tug1 is a long non-coding RNA mostly occurring in the retina and in the brain. It has been proposed to control cell growth by epigenetically down-regulating *CDKN1C*. In addition, Tug1 seems to predict a negative prognosis in gastric cancer [125]. These data indicate that lncRNAs regulate p57^Kip2^ at the cellular level probably acting in a phenotype-specific manner.

## 3. *CDKN1C* Expression and Human Diseases

Genetic and epigenetic disorders in the imprinted region 11p15 and *CDKN1C* mutations can lead to embryonic abnormalities, such as those occurring in Beckwith-Wiedmann syndrome (BWS; OMIM 130650), IMAGe syndrome (OMIM 614732) and Russell–Silver syndrome (RSS-OMIM 180860) and acquired diseases such as cancer.

### 3.1. Human Developmental Disorders

BWS, IMAGe syndrome and RSS are genetic diseases with different features, belonging to the group of congenital imprinting disorders. BWS has a prevalence of 1–5:10,000 live births and is characterized by overgrowth, tumor predisposition, abdominal wall defects and congenital malformations such as macroglossia, hemihyperplasia, hyperinsulinaemic hypoglycemia, ear anomalies, nephrologic and capillary malformations and organomegaly [128]. Epigenetic and genetic alterations in the imprinting cluster on chromosome 11p15.5 are responsible for up to 80% of BWS cases.

**Figure 2:**
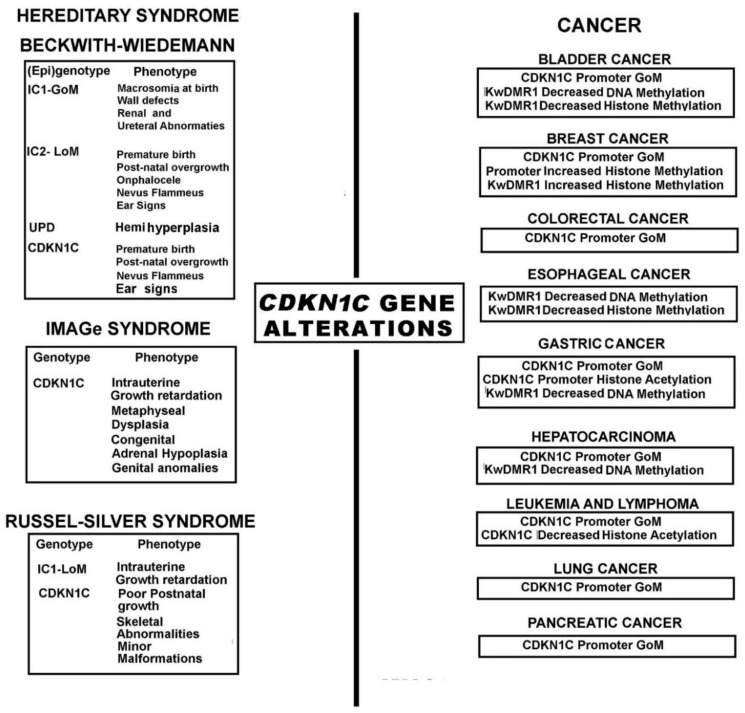
*CDKN1C* gene alterations in hereditary syndromes and human cancers. The figure reports on the left the Syndromes in which the *CDKN1C* gene is altered with the description of genotype alterations and main phenotypic features. On the right are reported the cancers showing *CDKN1C* genetic changes. IC1, ICR1 region; IC2, ICR2 region; GoM, gain of methylation; LoM, loss of methylation; UPD, Uniparental disomy.

They include epigenetic alterations such as methylation defects, specifically loss of methylation at IC2 regulatory region (IC2-LoM) and gain of methylation at IC1 (IC1-GoM), as well as genetic alterations including uniparental paternal disomy of the 11p15.5 locus (UPD), followed by a lower percentage of cases with microdeletion/duplications or point mutations involving either one of the two ICRs responsible for the locus imprinting region [129,130,131]. Of interest, in BWS patients without methylation defects, *CDKN1C* gene mutations are frequently noticed [132], reaching the 50–70% in familial BWS cases. Although with a lower occurrence, *CDKN1C* mutations are also reported in sporadic BWS cases and they have been identified as the causative genetic alterations [128,133]. Missense/nonsense mutations are reported along the entire sequence of the gene, leading to increased proliferation and risk of cancer [134,135]. Interestingly, these (epi) genotypes have been associated with specific phenotypes which discriminate mainly between overgrowth in pre- or postnatal age (Figure 2) [129].

Opposite to BWS, IMAGe (Intrauterine growth retardation, Metaphyseal dysplasia, congenital Adrenal hypoplasia, and Genital anomalies) syndrome is a rare condition (www.orpha.net) in which a cluster of CDKN1C missense mutations in the PCNA binding domain, result in growth inhibition) [136]. Interestingly, BWS and IMAGe, are characterized by loss of function and gain of function mutations of *CDKN1C,* respectively [136,137].

Imprinting alterations in the 11p15 region are also described in RSS, a disease (prevalence of 1–30:100,000 live birth) characterized by intrauterine growth retardation, very limited postnatal growth, skeletal abnormalities such as peculiar craniofacial characteristics and body asymmetry, and several minor malformations. Moreover, in RSS is also reported a maternal duplication in this region [138,139] and methylation alterations of imprinted genes on chromosome 7 [140]. Moreover, in one case of RSS a *CDKN1C* mutation affecting the PCNA binding domain has been found [141].

### 3.2. Human Cancers

Gene encoding cyclin-dependent inhibitors are frequently altered in human tumors. Among them, INK4 family represents the most clear paradigm [142,143]. The discovery of the involvement of p57^Kip2^ in BWS and in some human tumors suggests that p57^Kip2^, like p27^Kip1^, might also have a role in the process of carcinogenesis (Figure 2) [144]. Based on the roles played by the protein in the nuclear compartment, currently, it is considered a tumor suppressor; however, differently from p27^Kip1^ [143], somatic mutations have been rarely reported in tumors [145,146], underlining the importance of p57^Kip2^ expression control as the main cause of its altered levels in cancer. Particularly, a downregulation of *CDKN1C* is generally reported in cancer including gastric [147] and urothelial cancer [148,149], pancreatic adenocarcinomas [150], adrenocortical [151], lung [152], and breast cancer [153] as well as several leukemias [154]. Moreover, many authors have attributed to the p57^Kip2^ levels a value of prognostic marker since a decrease of its expression has been correlated to a poor prognosis [153,155] As described above, different epigenetic and genetic mechanisms can modulate the expression of *CDKN1C*. Essentially, loss of imprinting, DNA methylation and post-translational modifications of histones in the promoter region as well microRNAs downregulate *CDKN1C* in human cancers. Among them, the main cause of reduction of p57^Kip2^ in cancers is generally the increased methylation of the large CpG islands localized in the *CDKN1C* promoter [81,150,153,156]. Particularly, the promoter methylation of *CDKN1C* has been found critical in hematological malignancies such as acute lymphoblastic leukemia [157,158] and large B cell lymphoma [104]. Recently, Zohny and colleagues have proposed as diagnostic markers of breast cancer the expression levels of p21^CIP1/Waf1^ and p57^Kip2^ combined with the promoter methylation of *CDKN1C*, since they found a silenced expression of the two CKIs and a hypermethylation of *CDKN1C* promoter in more than 50% of the breast cancer specimens analyzed, together with no hypermethylation at promoter of p21^CIP1/Waf1^-coding gene [159]. Moreover, several miRNAs have been reported to control p57^Kip2^ mRNA levels. miR21 downregulates *CDKN1C* in prostate cancer [160], miR25 in gastric cancer and glioma [161,162] and miR92b in brain tumors [163]. Furthermore, miR221/222 are reported to reduce p57^Kip2^ and p27^Kip1^ expression in hepatocarcinoma [164], in glioblastoma [165], in oral cancer [166], in colorectal cancer [167] and B-cell malignancies EBV-associated [168] Experimental data confirmed, at least in ovarian cancers, the specific action of miR-221/222 on *CDKN1C* [169]. A further putative mechanism at the basis of *CDKN1C* down-regulation might be related to an increased rate of protein degradation, mainly due to the Skp2 overexpression, as frequently observed in human cancers. However, the relevance of Skp2-dependent degradation of p57^Kip2^ in carcinogenesis is still debated.

### 3.3. Other CDKN1C-Related Human Diseases

Gestational diseases, such as pre-eclampsia and intra-uterine growth restriction (IUGR), are also associated with altered p57^Kip2^ expression. Pre-eclampsia and IUGR are associated respectively with downregulation [170] and upregulation [171] of *CDKN1C*, underlining that a correct control of gene transcription is required for the proper development and progression of the pregnancy. p57^Kip2^, as reported above, is abundantly expressed in placental tissues, and, therefore, its dysregulated expression, in humans, is associated with placental mesenchymal and vascular proliferative disorders, such as placental mesenchymal dysplasia (PMD), and complete and partial hydatidiform moles [172,173].

Importantly, placentomegaly due to abnormal proliferation of extravillous trophoblasts, and accumulation of intervillous fibrinoid can be observed also in BWS syndrome [172,174].

PMD is a rare condition (0.02% pregnancies) associated with different fetal outcomes, ranging from structurally normal fetus/newborn (in most cases) to fetal and neonatal abnormalities, including those present in BWS, and mortality [175]. Indeed, PMD and BWS are associated in one-third of cases and paternal uniparental disomy at *IGF2* and *CDKN1C* locus has been proposed as the genetic link between them [176,177,178].

The identification of p57^Kip2^ as an important player in these placental diseases has led to the development of diagnostic procedures based on immunohistochemistry using anti-p57^Kip2^ antibodies and histological analysis for the characterization of hydatidiform moles and PMD, and their differential diagnosis [179,180,181].

Although some human diseases may represent very different clinical entities, some common pathways may be identified in their etiopathogenesis. This could be the case for cancer, metabolic diseases and related cardiovascular disease [182,183,184]. Indeed, p57^Kip2^, among others, may provide a similar origin for neoplastic proliferation and metabolic disorders. p57^Kip2^ is specifically expressed in the endocrine portion of the pancreas and particularly in β-cells where it is paternally imprinted [185]. In focal hyperinsulinism of infancy, a syndrome characterized by hyperinsulinemic hypoglycemia, p57^Kip2^ is not expressed within the focal adenomatous hyperplastic lesions. This missing expression is caused by somatic loss of heterozygosity and associated with increased proliferation of β-cells [185]. On this basis, p57^Kip2^ negative human pancreatic islets restored proper glucose control when transplanted into hyperglycemic, immunodeficient mice [186]. Interestingly, a gain-of-function mutant of p57^Kip2^ is associated with early-adulthood-onset diabetes, in addition to intrauterine growth restriction and short stature [187]. Not only glucose metabolism but also lipid metabolism could be affected by the genetic dosage of *CDKN1C*. In fact, a double dose of *Cdkn1c* promoted the brown adipose tissue development in a mouse model of the RSS [188]. Conversely, a loss-of-function mutation of p57^Kip2^, in the same model, hindered completely the formation of brown adipocytes [188].

The expression of p57^Kip2^ may also change according to genetic variations that do not pertain to *CDKN1C*, the p57^Kip2^-coding gene but are located in neighboring genomic sites. In particular, a mutation has been identified at the *KCNQ1* locus that increases the expression of p57^Kip2^ in mouse pancreatic islets by epigenetically modifying *Cdkn1c* [189]. Differently from the maternally transmitted *CDKN1C* diseases, this mutation is effective only when inherited from the paternal allele and, no matter which is the mechanism increasing the levels of p57^Kip2^ in the endocrine pancreas, β-cell mass is reduced [189].

Thus, it is conceivable that altering p57^Kip2^ expression could be a promising therapeutic strategy also in humans with type 2 diabetes and/or obesity. Indeed, oral administration of FTY720, a sphingosine 1-phosphate receptor agonist, normalizes glycemia in diabetic db/db mice by downregulating islet p57^Kip2^ and promoting β-cell regeneration [190]. However, body weight significantly increased in treated animals. Probably, tissue-specific targeting of p57^Kip2^ should be pursued, in order to avoid undesired effect in either pancreatic or adipose tissue when trying to manipulate p57^Kip2^ in one of the two tissues.

## 4. Future Directions

It has been clearly established that p57^Kip2^ plays pivotal and specific roles in human physiology that cannot be replaced by the other two members of its protein family, i.e., p27^Kip1^ and p21^Cip/WAF1^. As a matter of fact, *CDKN1C*-deleted mice show a very high percentage of mortality demonstrating that the protein is necessary for correct embryogenesis. Accordingly, in humans, three important syndromes, all showing an altered growth, are due to *CDKN1C* alterations. Particularly, the BWS is characterized by signs of overgrowth with infants considerably larger than normal and the IMAGe and RSS are both characterized by slow growth before and after birth and growth retardation.

It is well known that adult stem cells settle specific niches in different tissues and organs, thus supporting their repair/regeneration [57,191,192,193]. In this regard, p57^Kip2^ has been discovered as a very important factor [36,56,61,63,194].

The molecular mechanisms by which p57^Kip2^ is so important for a normal growth and tissue differentiation, are still not well understood. They refer, only in part, to the capability of the protein to modulate cyclin/CDK activity, a function that is played approximately by the first 100 residues of p57^Kip2^ where the KID is localized. On the other hand, numerous activities of CKI have been associated with the C-end region that includes the PAPA domain and the QT domain. These protein domains seem to be involved in the control of cell movement and the organization of mitotic spindle (via interaction with the cytoskeleton). Numerous pieces of evidence also suggest that p57^Kip2^ C-terminus participates in endoreduplication, apoptosis, autophagy, and senescence. Altogether these observations point to the definite identification of p57^Kip2^ interactors as a pivotal issue in the studies on CKI. Unfortunately, the protein belongs to the family of IUP, namely, proteins lacking a tertiary structure that fold upon binding. This structural feature allows p57^Kip2^ to have a large degree of plasticity and to interact with several different proteins. Although this is certainly a great advantage in terms of function, it results in remarkable difficulties in the precise definition of the mechanism of p57^Kip2^ action. An additional important aspect of p57^Kip2^ studies is the knowledge of processes that regulate its level. *CDKN1C* is subject to an epigenetic control and only the maternal allele is expressed. The gene lies in a locus, 11p15.5, that represents a major example of regional imprinting and that is strongly regulated by mechanisms acting in cis (i.e., directly on DNA structure) as well as in trans (namely via DNA methylation and histone acetylation). An additional level of intracellular control of p57^Kip2^ amount could be related to the protein degradation that involves, a not completely clarified, ubiquitination/proteasomal mechanism.

In conclusion, while the important role of p57^Kip2^ is clear, the details of its regulation and interactors appear enigmatic and intensive research and the development of novel cellular and animal models are required. This is particularly relevant in view of the plethora of p57^Kip2^ functions played in different tissues and distinct phenotypes both in normal and pathological conditions. It is conceivable that the elucidation of these aspects will provide important directions for human physiologic research and for the development of novel strategies for targeted therapy of several relevant human diseases.

## Abbreviations

BWS	Beckwith-Wiedmann syndrome
CSC	Cardia stem cell
CDK	Cyclin-dependent kinase
CKI	CDK inhibitor
DNMT	DNA methyltransferase
HSC	Hematopoietic stem cell
ICR	Imprinted control region
IGF2	Insulin-like gorwth factor
HDAC	histone deacetylase
IUGR	Intra-uterine growth restriction
IUP	Intrinsically unstructurated protein
KID	CDK binding/inhibitory domain
LSH	lymphoid-specific helicase
NLS	Nuclear localization signal
NSC	Neural stem cell
PCNA	Proliferating cell nuclear antigen
PMD	Placental mesenchymal dysplasia
PRC2	Polycomb repressive complex 2
p57^KO^	CDKN1C knockout
p57^KO^p27^KI^	CDKN1C knockout + CDKN1B knockin
RSS	Russell-Silver syndrome
Skp2	S-phase kinase-associated protein 2
SCF	Skp1/Cul1/F-box
TUG	Taurine upregulated gene 1
T310	Threonin 310
